# Supplementary material for: Polymorphisms in Pfkelch13 domains before and after the introduction of artemisinin-based combination therapy in Southwest Nigeria
Source: PLoS One. 2025 Mar 31;20(3):e0316479. doi: 10.1371/journal.pone.0316479 (PMC11957316; doi:10.1371/journal.pone.0316479)
Supplement: Supporting information 2 — (ZIP) [file pone.0316479.s002.zip › 141KN2F_PREMIX_Plate_KELCH2_C02.pdf]

Samples: 11967
Bases: 1008
Average spacing: 12

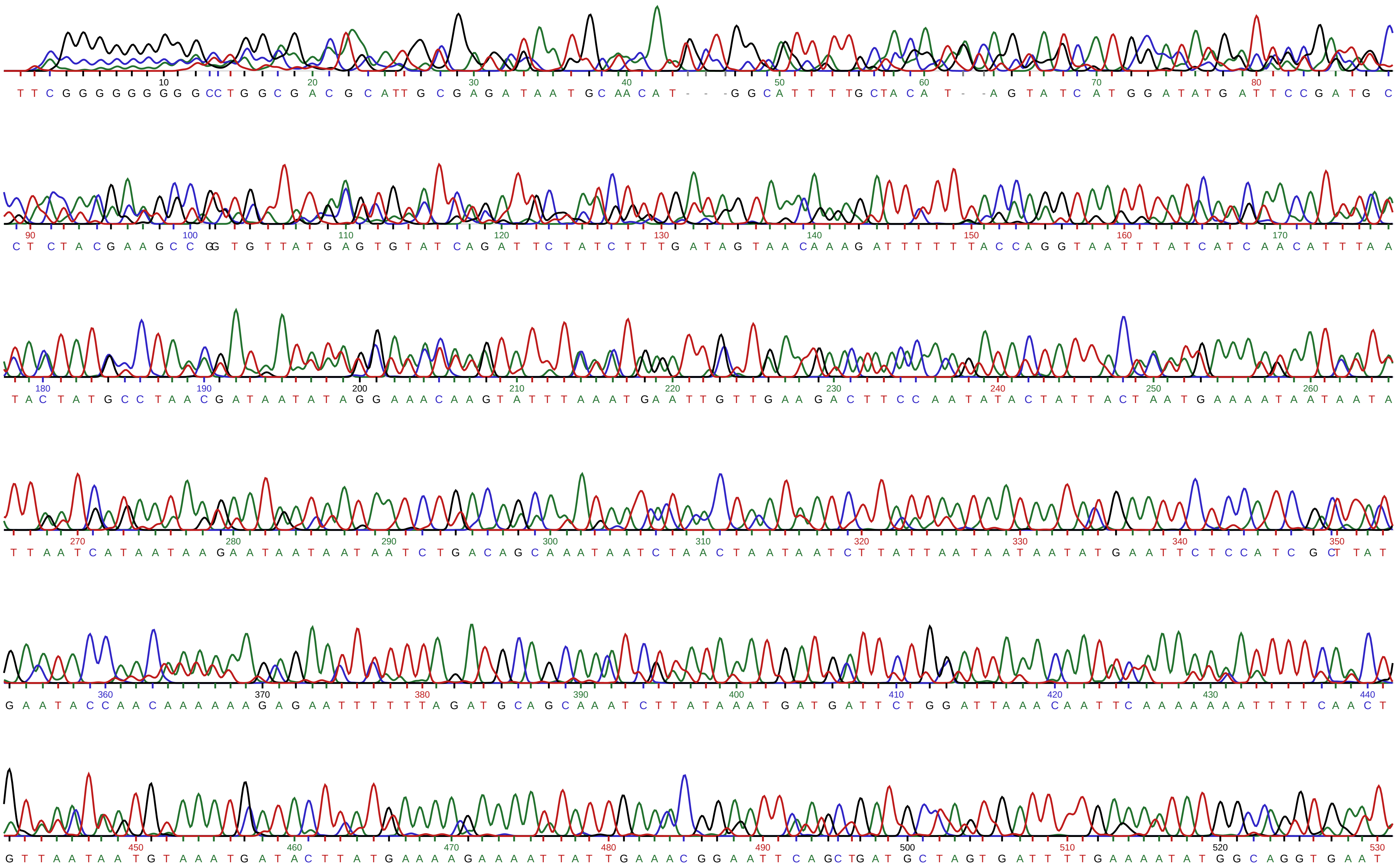

Samples: 11967
Bases: 1008
Average spacing: 12

8/17/2022

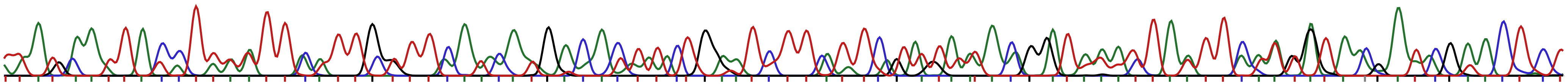

540 550 560 570 580 590 600 610 620

T T A T A A T T A C C T T A A T T C A T T G T T T C A A C A T G A C A C T T CT G A A T C T T A T T C G A T A A T A C G A T A A A T T A A T T C A A T A T A - G A T C G A A C T C T

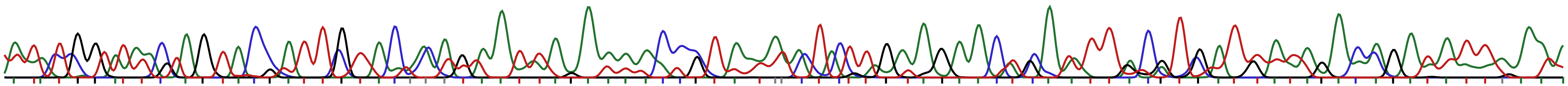

630 640 650 660 670 680 690 700

A TA T G G AT T C A G T A C G A T T G T A C - - - T A A T T A G A A A A C C G T A T -- C T CT T G T A G A A C T C A A T T A C G T G A T T A T A G A C A G A T A T T - A A A

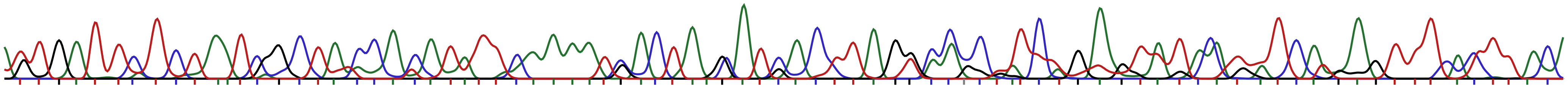

710 720 730 740 750 760 770 780 790

T T G A T TC T C T AAT C G C T A C C A C A T A T T C A A A A T G A C T A A G A T C A C T T A GG C C - C T AT C T G A G T A T C A T A T C A G A G T T T A C T T A C

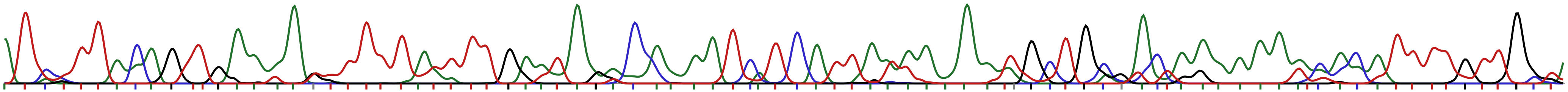

800 810 820 830 840 850 860 870 880

A T C T T T A C A G TT G A A A A - T T T T T A T T T T G A A T A G A C A A A A T CA T C A T T A A A A A A A T- G C T G C - A CT A A A A A A ATC A C A T T T T G T T G C T

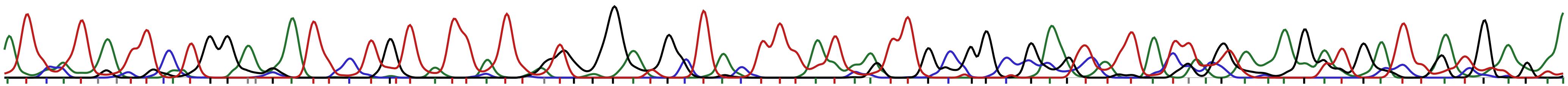

890 900 910 920 930 940 950 960

A T C A T - - T T CA T G G -- G A T T C T GT T A T T A T T - T G G G A T G C T A C T T T A T A A T T G C G G C G A G T T T A T - A G A A A G A T G A T T A T G A G A A

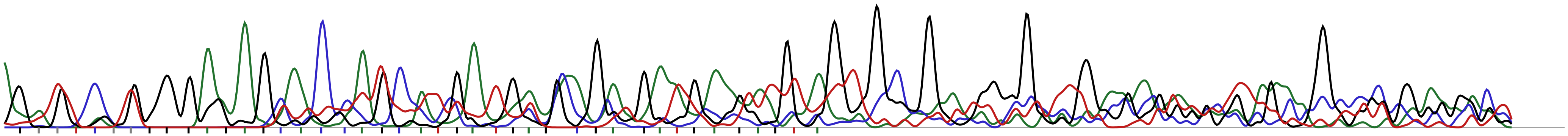

970 980 990 1000

G - - T C - - G G G A G A G C A C C A G C A T G A T G A G C G A G A T G A G A G T A
